# Supplementary figures and images for: Benfotiamine protects MPTP-induced Parkinson’s disease mouse model via activating Nrf2 signaling pathway
Source: PLoS One. 2024 Jul 23;19(7):e0307012. doi: 10.1371/journal.pone.0307012 (PMC11265681; doi:10.1371/journal.pone.0307012)

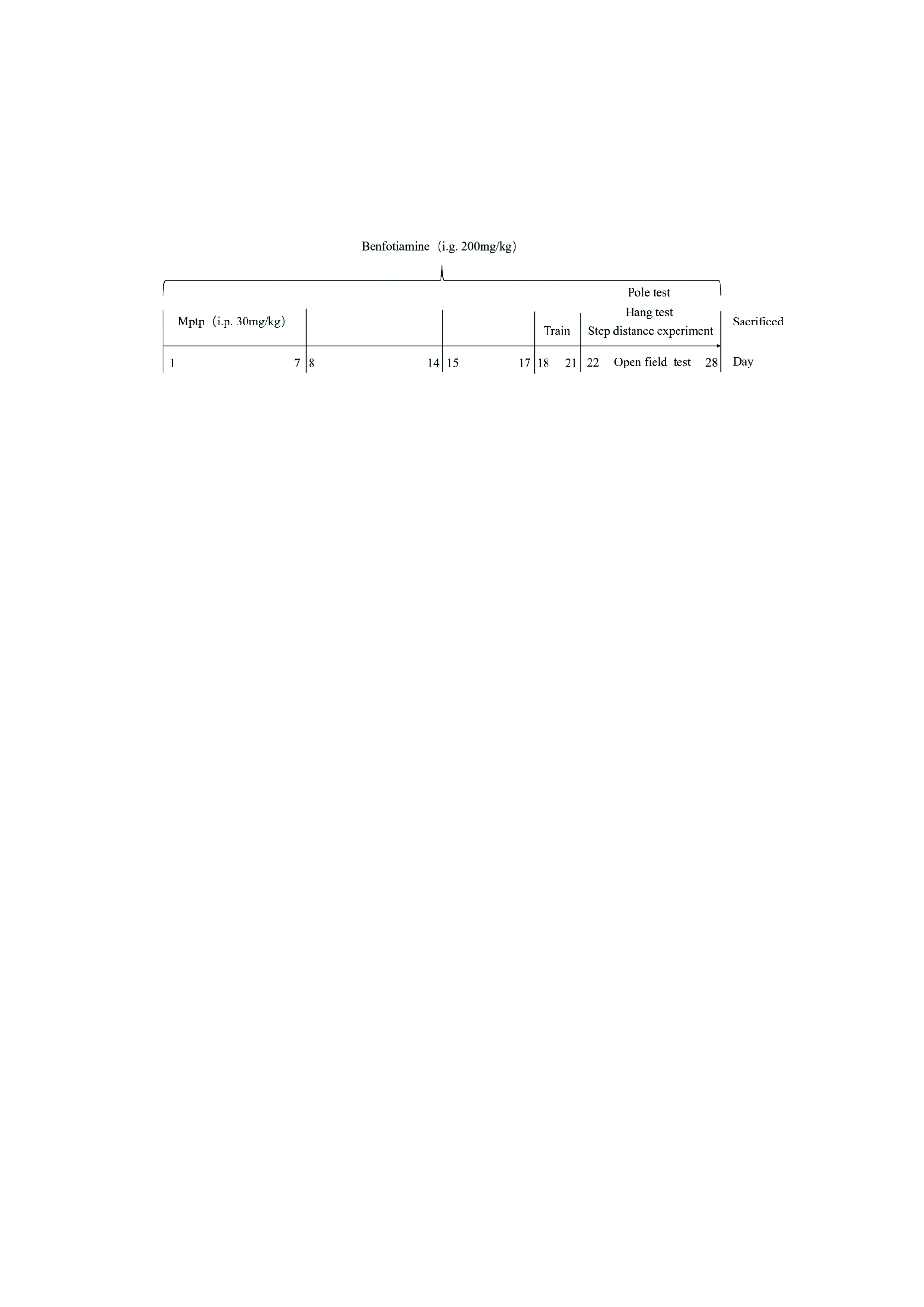

Supplement: S1 Fig — After a one-week acclimatization period, groups of mice received intraperitoneal injections of MPTP at a dose of 30 mg/kg for five consecutive days to induce a Parkinson’s disease model. The control group received the same amount of saline solution containing 0.9% NaCl. Mice were orally administered BFT at a dose of 200 mg/kg and 250 mg/kg, respectively, for 28 days, while the other group received intraperitoneal injections of MCC950 at a dose of 10 mg/kg for the same period of time. (TIF) [file pone.0307012.s001.tif]

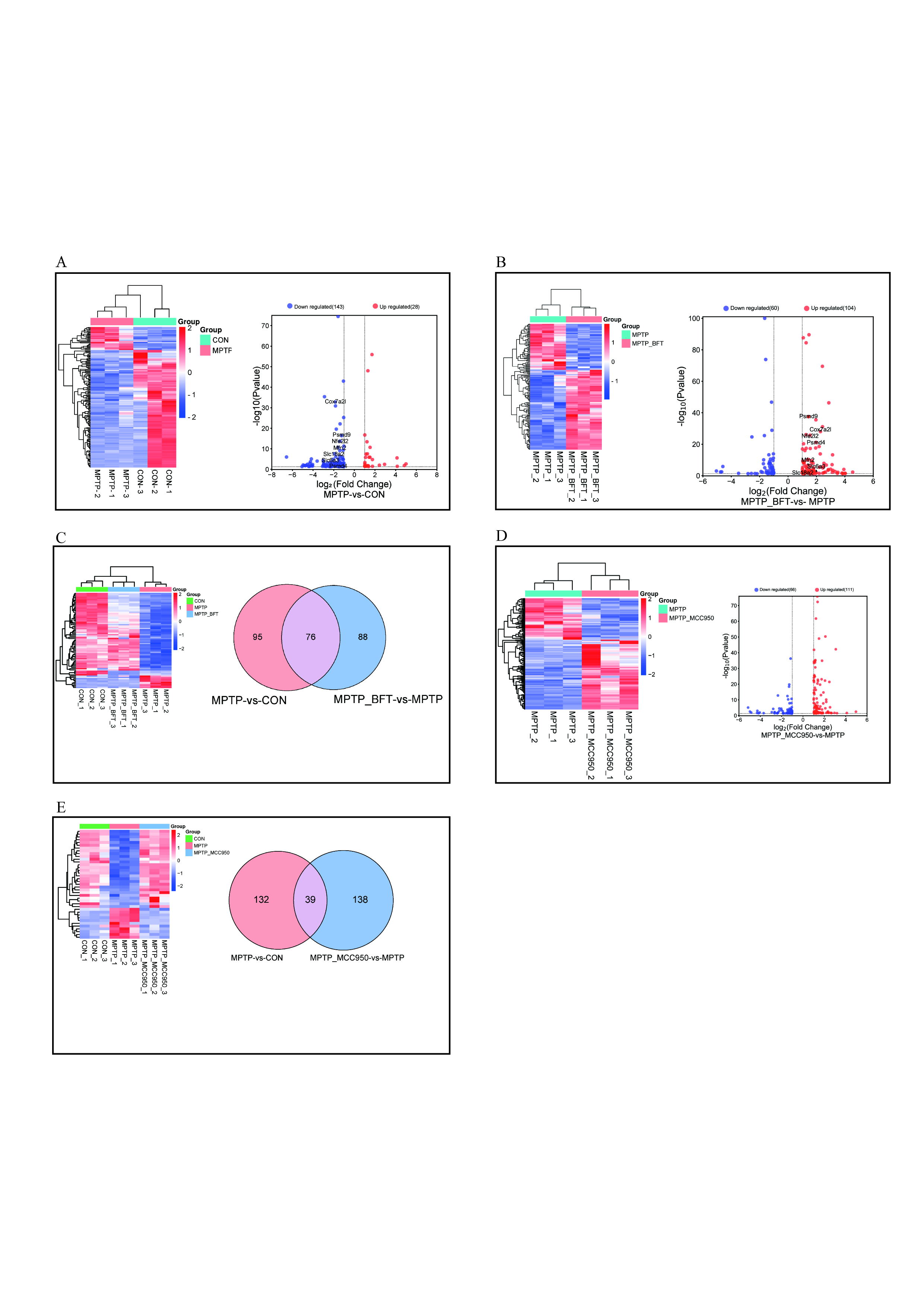

Supplement: S2 Fig — (A) Differential gene expression profiles between MPTP and CON comparison groups. (Left) Heatmap of relative expression of 171 differential genes; (Right) Differential gene volcano plot. (B) Differential gene expression profiles between MPTP_BFT and MPTP comparison groups. (Left) Heatmap of relative expression of 164 differential genes; (Right) Differential gene volcano plot. (C) Differential gene expression profiles in the intersecting region of MPTP vs CON and MPTP_BFT vs MPTP comparison groups. (Left) Heatmap of relative expression of 76 differential genes; (Right) Differential gene volcano plot. (D) MPTP_MCC950-vs-MPTP Differential Gene Expression Profile. (Left) Heat map of relative expression of 177 differential genes; (Right) Differential gene volcano map. (E) Differential gene expression profiles of MPTP vs CON and MPTP_MCC950-vs-MPTP shared changes. (Left) Heatmap of expression abundance of 39 co-altered differential genes; (Right) MPTP vs CON &MPTP_MCC950-vs-MPTP co-altered differential gene Venn diagram. Note: Differential gene criteria: p<0.05 and |Log2FC|≥1. red: up-regulated genes; blue: down-regulated genes. (TIF) [file pone.0307012.s002.tif]

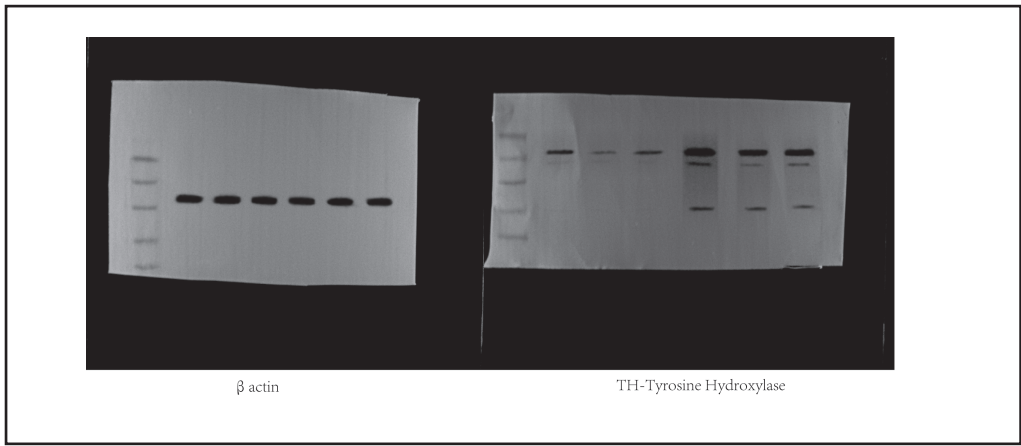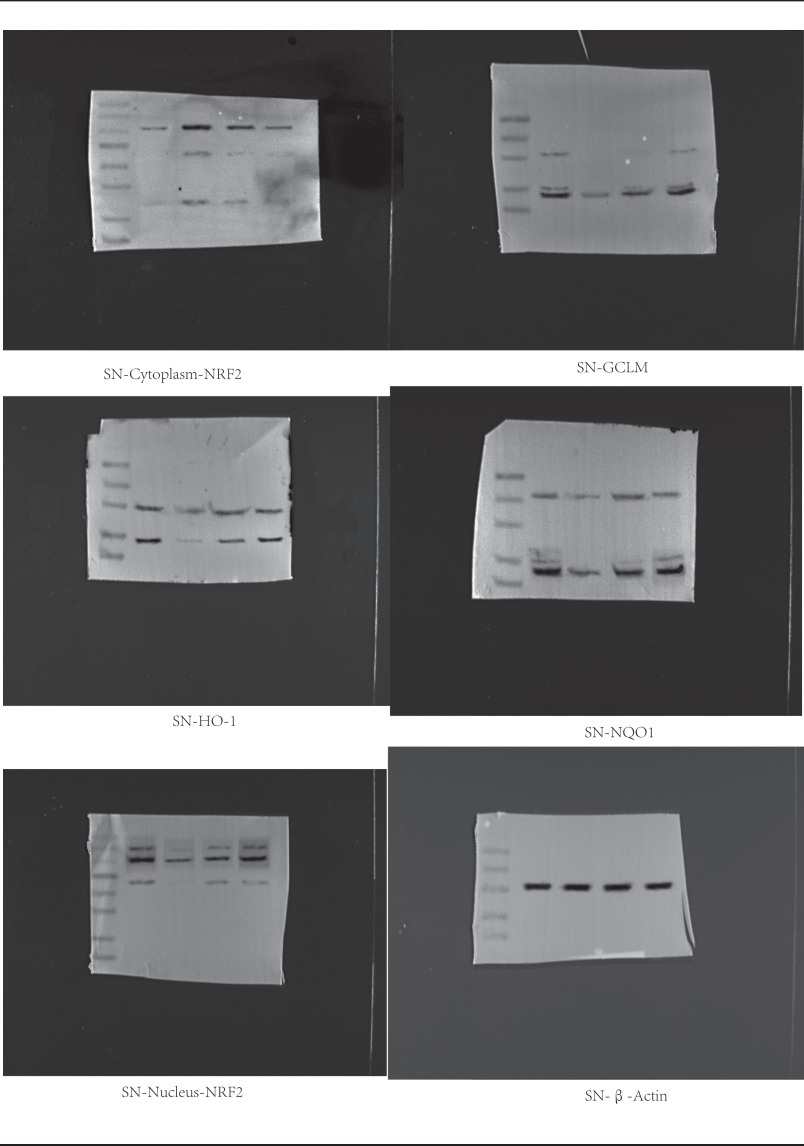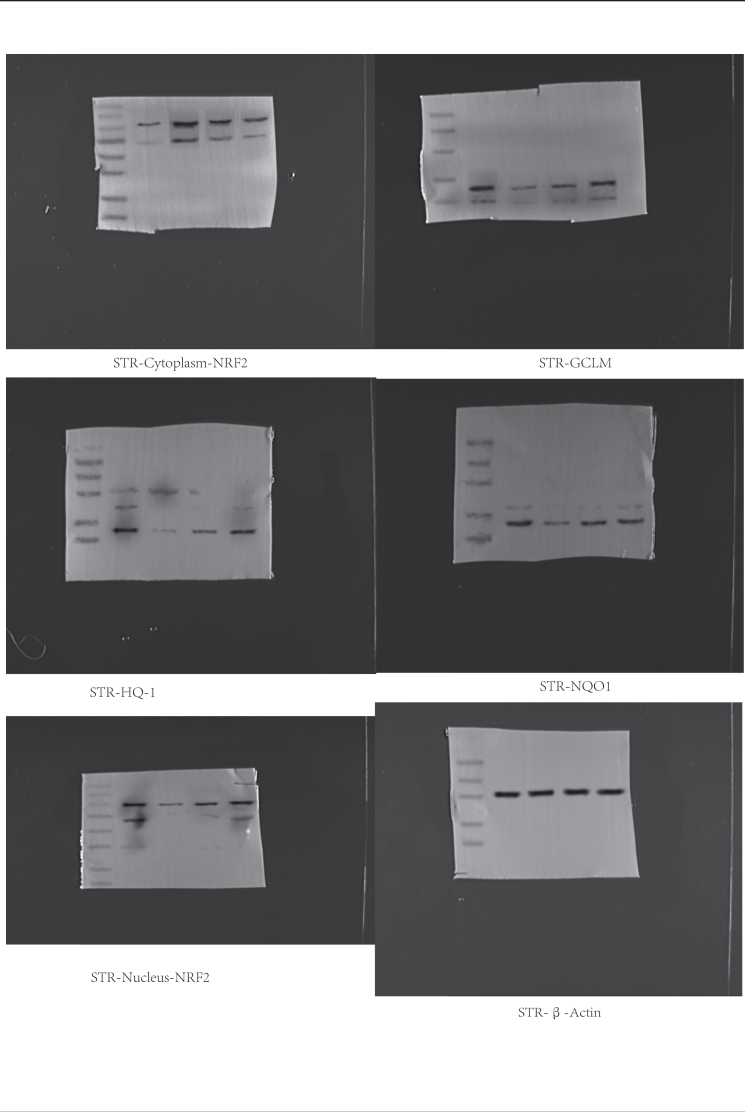

Supplement: S1 Raw images — (PDF) [file pone.0307012.s005.pdf]
