# Supplementary material for: Benfotiamine protects MPTP-induced Parkinson’s disease mouse model via activating Nrf2 signaling pathway
Source: PLoS One. 2024 Jul 23;19(7):e0307012. doi: 10.1371/journal.pone.0307012 (PMC11265681; doi:10.1371/journal.pone.0307012)
Supplement: Attachment — Submitted filename: plos one.docx [file pone.0307012.s006.docx]

**Authors Comment**

Author had written a good article entitled as “Benfotiamine Protects MPTP-Induced Parkinson's Disease Mouse Model via Activating Nrf2 Signaling Pathway”. Author aimed to examined the potential protective effects of BFT against damage to dopamine neurons in a PD animal model, as well as the underlying mechanisms. It is nicely written however, I have certain concern which, I have listed below:

1. Please mention which dose (200 or 250 mg/kg) of Benfotiamin (BFT), author has used in the experiment.
2. Figure S1 only showed the BFT 200 mg/kg response on mice. This makes incomplete experimentation.
3. If possible, please elaborate and pictorially depict the 200 mg/kg and 250 mg/kg, and MCC950 intraperitoneal injections, dosing response on mice in each experimentation (either on behavioral test or on molecular experimentation).
4. Please, elaborate BFT dose used in the present manuscript and why author has chosen the dose.
5. The present manuscript showed that mice in the MPTP group exhibited a loss in body weight upon MPTP injection, which gradually returned to normal levels after the cessation of MPTP injections. The mice in the BFT intervention group showed some increases in body weight compared to the MPTP group, but these changes were not statistically significant.
6. In the present study, the duration of BFT intervention in the study might not be sufficient to fully assess its long-term effects on PD progression. Longer intervention periods would be necessary to evaluate the sustained benefits of BFT treatment and its potential to slow down disease progression.
7. The present study suggests that BFT exerts its neuroprotective effects through various mechanisms such as Nrf2 pathway activation and antioxidant enhancement, the exact mechanisms underlying its action are not fully elucidated. Further research is needed to comprehensively understand how BFT functions at the molecular level and its potential interactions with other biological pathways.
8. If possible can author show, the degeneration of dopaminergic neurons by MPTP dosing and dopaminergic neuron regeneration by BFT using tyrosine hydroxylase staining.
