# Supplementary material for: Benfotiamine protects MPTP-induced Parkinson’s disease mouse model via activating Nrf2 signaling pathway
Source: PLoS One. 2024 Jul 23;19(7):e0307012. doi: 10.1371/journal.pone.0307012 (PMC11265681; doi:10.1371/journal.pone.0307012)
Supplement: Attachment — Submitted filename: Response to Reviewers.docx [file pone.0307012.s007.docx]

Reviewer #1: Authors Comment
Author had written a good article entitled as “Benfotiamine Protects MPTP-Induced Parkinson's Disease Mouse Model via Activating Nrf2 Signaling Pathway”. Author aimed to examined the potential protective effects of BFT against damage to dopamine neurons in a PD animal model, as well as the underlying mechanisms. It is nicely written however, I have certain concern which, I have listed below:

1.Please mention which dose (200 or 250 mg/kg) of Benfotiamin (BFT), author has used in the experiment.
2.Figure S1 only showed the BFT 200 mg/kg response on mice. This makes incomplete experimentation.
3.If possible, please elaborate and pictorially depict the 200 mg/kg and 250 mg/kg, and MCC950 intraperitoneal injections, dosing response on mice in each experimentation (either on behavioral test or on molecular experimentation).
4.Please, elaborate BFT dose used in the present manuscript and why author has chosen the dose.
5.The present manuscript showed that mice in the MPTP group exhibited a loss in body weight upon MPTP injection, which gradually returned to normal levels after the cessation of MPTP injections. The mice in the BFT intervention group showed some increases in body weight compared to the MPTP group, but these changes were not statistically significant.
6.In the present study, the duration of BFT intervention in the study might not be sufficient to fully assess its long-term effects on PD progression. Longer intervention periods would be necessary to evaluate the sustained benefits of BFT treatment and its potential to slow down disease progression.
7.The present study suggests that BFT exerts its neuroprotective effects through various mechanisms such as Nrf2 pathway activation and antioxidant enhancement, the exact mechanisms underlying its action are not fully elucidated. Further research is needed to comprehensively understand how BFT functions at the molecular level and its potential interactions with other biological pathways.
8.If possible can author show, the degeneration of dopaminergic neurons by MPTP dosing and dopaminergic neuron regeneration by BFT using tyrosine hydroxylase staining.

Reviewer #2: My comments on the manuscript entitled “Benfotiamine Protects MPTP-Induced Parkinson's Disease Mouse Model via Activating Nrf2 Signaling Pathway” are as follows.
How does BFT intervention impact motor deficits in the PD mouse model, as assessed by the pole test, hang test, gait analysis, and open field test? Are there specific improvements noted in certain types of motor functions over others?
Can you elaborate on the methods used to evaluate the extent of damage to dopaminergic neurons in the substantia nigra and striatum following MPTP-induced injury, particularly regarding the techniques of Immunohistofluorescence, Nissl staining, and Western blot analysis of Tyrosine Hydroxylase (TH)?
What specific changes in dopamine (DA) levels and its metabolites were observed following BFT intervention, as measured by High Performance Liquid Chromatography (HPLC)? Were these changes statistically significant, and how do they correlate with the observed neuroprotective effects?
Regarding the RNA-seq and bioinformatics analysis, what were the key pathways and genes affected by BFT intervention in the substantia nigra tissues? How do these findings compare with those from treatment with the NLRP3 inhibitor MCC950, and what implications do these differences have for Parkinson's disease research?
Could you provide more detail on the findings related to the activation of the Nrf2 pathway by BFT intervention? Specifically, how was the movement of Nrf2 to the nucleus assessed, and what were the observed changes in the expression of downstream antioxidant genes and enzymes in the substantia nigra and striatum?
In what ways did BFT treatment affect oxidative damage markers such as MDA content, GSH activity, and SOD activity in the substantia nigra and striatum? Were these changes consistent with the observed neuroprotective effects, and do they provide further insight into the mechanisms of BFT's action in PD?
Given the findings of this study, what are the potential implications for the development of BFT as a therapeutic agent for Parkinson's disease in humans? What additional preclinical or clinical studies are needed to further investigate its efficacy and safety profile in PD patients?
How does the global prevalence of Parkinson's Disease (PD) in 2016 compare to that of 1990, according to the provided data?
What are the primary clinical manifestations of PD, and which brain regions are predominantly affected by its pathological changes?
What are the limitations of current treatments for PD, such as dopamine replacement therapy and Deep Brain Stimulation?
How does thiamine (vitamin B1) potentially influence the onset and development of PD, based on the described research findings?
What are the advantages of using benfotiamine (BFT) over thiamine in terms of its bioavailability and ability to traverse the blood-brain barrier?
Similar to Benfotiamine, Mucuna pruriens, Ursolic acid and Chlorogenic acid also exhibited similar kind of therapeutic activity in MPTP intoxicated mouse model. Cite original article for the same and correlate with your own outcomes.
Can you summarize the findings regarding the neuroprotective effects of BFT in both Alzheimer's disease and PD, as described in the text?
How does 1-methyl-4-phenyl-1,2,3,6-tetrahydropyridine (MPTP) induce damage in dopaminergic neurons, and what model of PD does it represent?
What were the main results of the study investigating the protective effects of BFT on motor function and dopaminergic neurons in the MPTP mouse model of PD?
What is MCC950, and how does it relate to the NOD-like receptor pyrin domain-containing protein 3 (NLRP3)?
How did the study utilize RNA sequencing technology to investigate the protective mechanisms of BFT and MCC950 on dopaminergic neurons, and what were the key findings regarding their potential synergistic effects?
Provide exact p value for all your histograms.
Positive control is missing in your experiment.
Provide 4x, 10x and other higher magnification images for all your immunoflourescence.
Provide complete western blot, not the cut one.
Improve clarity and resolution of all the western blot in your manuscript.

Dear Reviewer #1,

Thank you for your constructive comments on our manuscript entitled "Benfotiamine Protects MPTP-Induced Parkinson's Disease Mouse Model via Activating Nrf2 Signaling Pathway." We have taken your suggestions seriously and have made the following revisions:

1. We have specified the doses of Benfotiamine (BFT) used in our experiments. Both 200 mg/kg and 250 mg/kg doses were utilized for the study.
2. We acknowledge the oversight in Figure S1 and have now included the response for the 250 mg/kg BFT dose group to provide a complete picture of the experimental outcomes.
3. We have expanded the manuscript to include detailed descriptions and graphical representations of the dose-response effects of both 200 mg/kg and 250 mg/kg BFT, as well as MCC950 injections on mice across behavioral and molecular experiments.
4. The rationale for the chosen BFT doses has been elaborated upon in the manuscript, providing a clear explanation for our selection.
5. We have further discussed the observed body weight changes in the BFT intervention group and acknowledged the limitations in statistical significance. Future studies will consider employing more sensitive methods for body weight monitoring.
6. We agree that the duration of BFT intervention may not fully assess long-term effects. We have addressed this in the discussion and suggest the necessity of longer-term studies to evaluate sustained benefits.
7. The mechanisms underlying BFT's neuroprotective effects have been further explored in the revised manuscript. We have proposed future research directions to better understand BFT's molecular functions and interactions with biological pathways.
8. We have included additional data using tyrosine hydroxylase staining to visualize the degeneration and potential regeneration of dopaminergic neurons following MPTP exposure and BFT treatment, respectively.

Dear Reviewer #2,

Thank you for your insightful comments on our manuscript. We have made the following revisions in response to your queries:

1. The impact of BFT intervention on motor deficits has been detailed, with specific improvements in motor functions discussed.
2. We have expanded the methods section to provide a more thorough explanation of the techniques used to evaluate dopaminergic neuron damage, including Immunohistofluorescence, Nissl staining, and Western blot analysis of Tyrosine Hydroxylase (TH).
3. Detailed changes in dopamine (DA) levels and metabolites following BFT intervention, along with their statistical significance and correlation to neuroprotective effects, are now presented.
4. Key pathways and genes affected by BFT intervention have been identified and compared with those from MCC950 treatment, discussing the implications for Parkinson's disease research.
5. We have provided more details on the activation of the Nrf2 pathway by BFT, including assessment methods for Nrf2 nuclear translocation and changes in downstream antioxidant gene and enzyme expression.
6. The effects of BFT treatment on oxidative damage markers have been discussed, along with their consistency with neuroprotective effects and insights into BFT's mechanisms of action in PD.
7. The potential implications for developing BFT as a therapeutic agent for PD have been explored, along with suggestions for additional preclinical and clinical studies.
8. Data comparing the global prevalence of Parkinson's Disease (PD) in 2016 to 1990 has been included.
9. The primary clinical manifestations of PD and the predominantly affected brain regions have been detailed.
10. Limitations of current PD treatments, such as dopamine replacement therapy and Deep Brain Stimulation, have been discussed.
11. The potential influence of thiamine (vitamin B1) on the onset and development of PD has been reviewed.
12. The advantages of using benfotiamine (BFT) over thiamine in terms of bioavailability and blood-brain barrier penetration have been highlighted.
13. We have cited original articles for Mucuna pruriens, Ursolic acid, and Chlorogenic acid and correlated their therapeutic activities with our outcomes.
14. A summary of the neuroprotective effects of BFT in both Alzheimer's disease and PD has been provided.
15. The mechanism of MPTP-induced damage in dopaminergic neurons and the PD model it represents has been explained.
16. Main results of the study on the protective effects of BFT on motor function and dopaminergic neurons in the MPTP mouse model of PD have been discussed.
17. MCC950 and its relation to the NOD-like receptor pyrin domain-containing protein 3 (NLRP3) have been described.
18. The use of RNA sequencing technology to investigate the protective mechanisms of BFT and MCC950, and the key findings regarding their potential synergistic effects, have been detailed.
19. Exact p-values for all histograms have been provided.
20. A positive control has been added to the experiments.
21. Higher magnification images for all immunofluorescence have been included.
22. Complete Western blot images have been provided instead of the cut ones.
23. The clarity and resolution of all Western blots in the manuscript have been improved.

We appreciate the opportunity to improve our manuscript and believe that these revisions have strengthened our work. We look forward to your further feedback.

Best regards,

Yu Wang

Corresponding author: Yu Wang

Emial: yw4d@hotmail.com
