# Supplementary material for: Benfotiamine protects MPTP-induced Parkinson’s disease mouse model via activating Nrf2 signaling pathway
Source: PLoS One. 2024 Jul 23;19(7):e0307012. doi: 10.1371/journal.pone.0307012 (PMC11265681; doi:10.1371/journal.pone.0307012)
Supplement: Attachment — Submitted filename: rebuttal letter.docx [file pone.0307012.s008.docx]

Dear Editor and Reviewer #3,

We would like to express our sincere gratitude for the opportunity to revise our manuscript and for the insightful comments provided. We have taken the suggestions seriously and have made substantial revisions to address the concerns raised. Below, we provide a point-by-point response to the reviewer's comments.

1,Comment:** The study lacks a clear rationale.

**Response:** We acknowledge that the rationale for our study could have been more explicit. To address this, we have added a section in the introduction that clearly outlines the gaps in the current literature that our study aims to fill. Specifically, we have included a discussion on the limited understanding of BFT's role in the Nrf2-ARE signaling pathway in the context of PD, which we believe provides a strong rationale for our research.

2,Comment:** The compound has already been documented to have a neuroprotective effect against MPTP-induced Parkinson's disease in rats.

**Response:** We appreciate the reviewer's reference to the previous study (PMID: 38043777). While it is true that BFT has been reported to have neuroprotective effects, our study offers novel insights by focusing on the Nrf2-mediated antioxidant mechanisms and gene expression modulation in a PD mouse model. We have now included a comparative analysis with the previous study to highlight the differences and contributions of our research.

3,Comment:** The discussion section should elaborate on the behavioral experimental results and their connection to the changes observed after BFT supplementation.

**Response:** We agree with the reviewer that a more detailed discussion of the behavioral results is necessary. We have expanded the discussion section to support the correlation between behavioral improvements and the neuroprotective effects of BFT.

4,Comment:** The current discussion is inadequate and does not effectively support the results.

**Response:** We have revised the discussion section to provide a more comprehensive interpretation of our results. We have included a critical analysis of how our findings fit into the broader context of PD research and have addressed the potential implications of our results for future therapeutic strategies.

5,Comment:** The authors are advised to go through the following articles.

**Response:** We thank the reviewer for the additional references provided. We have reviewed these articles and have incorporated relevant findings and discussions into our revised manuscript. Specifically, we have expanded our discussion on the role of Nrf2 in neuroprotection and have included a comparison with other antioxidants and neuroprotective agents mentioned in the referenced articles.

In conclusion, we believe that our revisions have significantly strengthened the manuscript. We have provided a clearer rationale, addressed the existing literature more effectively, and enhanced the discussion to better support our results. We hope that our revisions meet the expectations of the reviewers and the journal.

Sincerely,

Yu Wang, yw4d@hotmail.com.

Department of Neurology, The First Affiliated Hospital of Anhui Medical University. Jixi Road 218, Shushan District, Hefei, Anhui Province 230000, People’s Republic of China.
